# Endogenous zinc nanoparticles in the rat olfactory epithelium are functionally significant

**DOI:** 10.1038/s41598-020-75430-w

**Published:** 2020-10-28

**Authors:** Melissa Singletary, June W. Lau, Samantha Hagerty, Oleg Pustovyy, Ludmila Globa, Vitaly Vodyanoy

**Affiliations:** 1 Department of Anatomy, Physiology and Pharmacology, Auburn University College of Veterinary Medicine, Auburn, AL USA; 2 Canine Performance Sciences Program, Auburn University College of Veterinary Medicine, Auburn, AL USA; 3 Material Measurement Laboratory, National Institute of Standards and Technology, Gaithersburg, MD USA

**Keywords:** Biophysics, Neuroscience, Physiology, Nanoscience and technology, Physics

## Abstract

The role of zinc in neurobiology is rapidly expanding. Zinc is especially essential in olfactory neurobiology. Naturally occurring zinc nanoparticles were detected in olfactory and nasal respiratory epithelia and cilia in animals. The addition of these nanoparticles to a mixture of odorants, including ethyl butyrate, eugenol, and carvone, considerably increased the electrical responses of the olfactory sensory receptors. Studies of these nanoparticles by ransmission electron microscopy (TEM) and selected area electron diffraction revealed metal elemental crystalline zinc nanoparticles 2–4 nm in diameter. These particles did not contain oxidized zinc. The enhancement of the odorant responses induced by the endogenous zinc nanoparticles appears to be similar to the amplification produced by engineered zinc nanoparticles. Zinc nanoparticles produce no odor response but increase odor response if mixed with an odorant. These effects are dose-dependent and reversible. Some other metal nanoparticles, such as copper, silver, gold, and platinum, do not have the effects observed in the case of zinc nanoparticles. The olfactory enhancement was observed in young and mature mouse olfactory epithelium cultures, in the dissected olfactory epithelium of rodents, and in live conscious dogs. The physiological significance of the detected endogenous metal nanoparticles in an animal tissue has been demonstrated for the first time. Overall, our results may advance the understanding of the initial events in olfaction.

## Introduction

The process of odor perception, i.e., olfaction, begins with sniffing, which brings the odorant molecules into the nose and delivers them to the mucus layer covering the olfactory epithelium. The binding of the odorant by a receptor protein in the olfactory cilia initiates an intracellular cascade of signal transduction events involving the G-protein-dependent production of the second messenger molecules and leading to the opening of the ion channels to induce the ion currents. This process triggers an action potential in the olfactory sensory receptor neurons that projects directly to the olfactory bulb. The signal is then transmitted to the other parts of the brain for recognition and interpretation^1^.

We have previously reported that the zinc metal nanoparticles at very low concentrations enhance olfactory responses to an odorant. The addition of these particles to an odorant increases electrical responses of the odorant receptors by up to threefold^2^. Subsequent studies have demonstrated that zinc nanoparticles function at the olfactory receptor level in the initial events of olfaction^3^, and a single metal nanoparticle binds two receptor molecules to form a dimer^4^. This olfactory enhancement was observed in young and mature mouse olfactory epithelial cultures, dissected rodent olfactory epithelium^2,3,5,6^, and live conscious dogs^7,8^.

Zinc plays a considerable role in neurobiology^9^. In most cases, the distribution of zinc in the brain was studied by the methods that cannot discriminate between zinc ions and zinc metal particles^10^. We have demonstrated that engineered zinc, which enhanced olfactory responses to the odorant, exists in a nonoxidized elemental metal state and comprises round crystalline clusters of zinc atoms ~ 1.2 nm in diameter^5^.

Ultrafiltration of human and animal blood through 30 and 5 kDa filters resulted in the detection of zinc, copper, and iron metals in the filtrates by energy-dispersive X-ray spectroscopy (EDS) and inductively coupled plasma-atomic emission spectrometry. Transmission electron microscopy (TEM) showed that the filtrate contained crystalline metallic nanoparticles 1–2 nm in diameter. Selected area electron diffraction (SAD) patterns revealed the presence of copper and iron nanoparticles^11^. Subsequently, these filtrates were shown to contain 1–2 nm metallic nanoparticles of various metals, including zinc, that were capable to enhance olfactory responses similar to the engineered zinc nanoparticles^2^.

The goal of the present study was to determine whether endogenous zinc nanoparticles are present in the olfactory epithelium or cilia and whether the enhancement of olfactory responses by zinc nanoparticles is physiologically significant.

## Results

### Endogenous zinc nanoparticles enhance olfactory responses to an odorant

**Figure 1:**
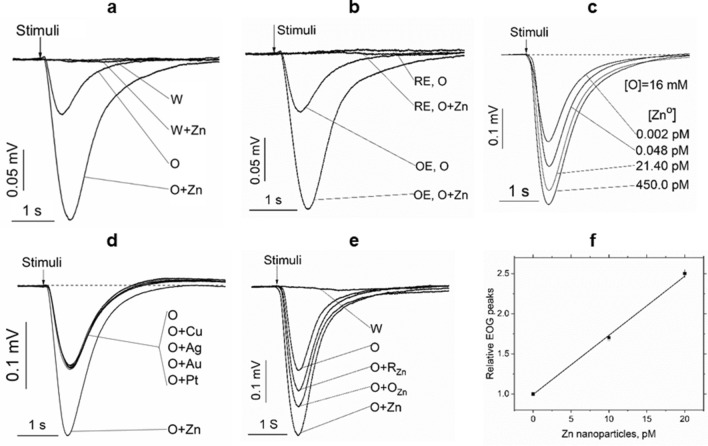
Electrical responses of olfactory and respiratory epithelia to an odorant and metal nanoparticles. a. EOG traces induced by water vapor (W), water vapor with 10 pM zinc nanoparticles (W + Zn), 16 mM odorant (O), and 16 mM odorant with 10 pM engineered zinc nanoparticles. The figure shows a representative of 220 experimental EOG traces. b. Electrical responses of olfactory (OE) and respiratory (RE) epithelia to 16 mM odorant (OE, O and RE, O, respectively) and to 16 mM odorant + 10 pM engineered zinc particles (OE, O + Zn and RE, O + Zn, respectively). The figure shows a typical representative of 120 traces. c. EOG recordings induced by 16 mM odorant + engineered zinc nanoparticles at concentrations from 0.002 pM to 450 pM. The figure shows a representative of 260 EOG traces. d. EOG induced by 16 mM odorant (O) and odorant + 20 pM of Cu, Ag, Au, Pt, or Zn nanoparticles. A set of traces is shown representative of 30 EOG responses. e. EOG responses to water vapor (W), 1.6 mM odorant (O), 1.6 mM odorant + zinc nanoparticles obtained from respiratory cilia (O + R_Zn_) or olfactory cilia (O + O_Zn_), or 20 pM of engineered zinc nanoparticles (O + Zn). The figure is a representative of 120 EOG traces. f. Relative EOG peaks as a function of concentration of engineered zinc nanoparticles (ratio of EOG peak induced by an odorant + zinc to the peak generated by an odorant). The points are experimental data, and the line is a linear fit (R-square = 0.997; intercept = 0.99 ± 0.02; slope = 73.6 ± 2.7 1/pM). The odorant-induced peaks were calculated using 48 EOG traces, and the peaks were induced by an odorant + 10 pM zinc and an odorant + 20 pM zinc; the data of two independent sets of 24 EOG traces were used for calculations.

When a short pulse of an odorant was delivered to the olfactory epithelium, the olfactory receptors in the cilia of the olfactory sensory neurons initiate the production of the generator potentials. The measuring electrode collects a sum of these potentials from a set of the receptors as an electroolfactogram (EOG) and records it as a function of time. The vertebrate EOGs typically have a peak shape and last for approximately one second, and their amplitude depends on the set of the receptors and odorant concentration^12^. Zinc nanoparticles produce no odor effects alone but increase odor response if mixed with an odorant (Fig. 1a).

In contrast, the respiratory epithelium does not respond to an odorant or an odorant with engineered zinc nanoparticles (Fig. 1b). The effects of engineered zinc nanoparticles are dose-dependent and effective across a large range of zinc concentrations (Fig. 1c). Other tested metal nanoparticles, such as copper, gold, and silver, do not enhance the olfactory response (Fig. 1d).

Response enhancement induced by the addition of zinc nanoparticles obtained from the olfactory and respiratory epithelia to an odorant was compared to that of the engineered nanoparticles (Fig. 1e). Zinc nanoparticles obtained from the olfactory and respiratory cilia considerably enhanced the response to an odorant. To estimate the concentration of zinc nanoparticles in the filtrates from the olfactory and respiratory epithelia and cilia, a calibration curve was obtained by recording the responses to an odorant in the presence of the known concentrations of engineered zinc nanoparticles (Fig. 1f). The concentrations of zinc nanoparticles detected in the olfactory and respiratory filtrates were 0.27 ± 0.05 nM and 0.11 ± 0.05 nM, respectively. The concentrations of zinc nanoparticles detected in the olfactory and respiratory cilia filtrates were 0.25 ± 0.05 nM and 0.36 ± 0.05 nM, respectively. To convert the filtrate concentrations into the epithelial and cilia concentrations, the relative volumes of the epithelium and cilia have to be estimated. The main geometrical features of the olfactory and respiratory epithelium and cilia according to the data of the literature are shown in Table 1.

**Table 1:** Characterization of rat olfactory and nasal respiratory epithelia.

Feature	Olfactory epithelium	Ref.	Respiratory epithelium	Ref.
Surface area, mm^2^	675 ± 43	^65^	623 ± 14	^65^
Thickness, µm	60 ± 5	^66^	27 ± 3	^67^
Density of ciliated cells, 1/cm^2^	(6.0 ± 0.3) × 10^7^	^66^	(1.2 ± .06) × 10^7^	^68^
Number of cilia per cell	25 ± 5	^69^	11 ± 3	^12^
Cilia diameter, µm	0.20 ± 0.04	^69^	0.27 ± 0.04	^69^
Cilia length, µm	12.5 ± 2.5	^69^	3.2 ± 0.1	^68^

The data of Table 1 indicate that the volume of the olfactory and respiratory epithelia can be calculated according to the equation V = A × T, where A and T are the epithelial surface area and thickness, respectively. The volume of the olfactory and respiratory cilia is given by the equation V_c_ = (πd^2^/4) × l × A × D × n, where d is the diameter of a cilia, l is the length of a cilia, A is epithelial area, D is the density of ciliated cells, and n is the number of cilia per cell (Supplementary Fig. S5). The volume of the olfactory and respiratory epithelia and cilia and the relative volumes calculated based on the data of the literature are shown in Table 2.

**Table 2:** Volumes of epithelia and cilia.

Epithelium/cilia	Olfactory epithelium	Respiratory epithelium
Epithelium, V_e_, cm^3^	(4.1 ± 0.6) × 10^−2^	(1.7 ± 0.2) × 10^−2^
Cilia, V_c_, cm^3^	(4.0 ± 04) × 10^−3^	(3.4 ± 0.2) × 10^−4^
V_c_/V_e_, %	10.0 ± 2.0	2.0 ± 0.3

Table 2 shows that the volume of the cilia of the olfactory sensory neurons is 10% of the volume of the olfactory epithelium, and the volume of the cilia of the ciliated respiratory cells is only 2% of the volume of the respiratory epithelium.

In our experiments, the weight of the olfactory and respiratory epithelia (m) used to prepare the filtrates was measured. The volumes of the experimental tissue can be calculated from the weight according to the equation V = m/ρ, where m is tissue weight (g) and ρ is the specific gravity in g/cm^3^. Estimation of the contents of the cilia as 20% protein (1.4 g/cm^3^), 5% carbohydrates (1.6 g/cm^3^), and 75% lipids/water (1.0 g/cm^3^) gives the averaged uniform specific gravity of the cilia of 1.11 g/cm^3^ ^13^. The specific gravity of oral mucosa and brain neurons is 1.036 g/cm^3^ ^14^ and 1.050 g/cm^3^ ^15^, respectively. If the specific gravities of the olfactory and respiratory epithelia are not substantially different from those of the brain neurons and oral mucosa, the specific gravity of the tissues used in our experiments is assumed to equal approximately 1 g/cm^3^.

Experimental weight of the samples of the olfactory and respiratory epithelia was 90 ± 5 mg and 60 ± 5 mg, respectively. Similarly, the volumes of the olfactory and respiratory epithelia are (9.0 ± 0.5) × 10^−2^ cm^3^ and (6.0 ± 0.4) × 10^−2^ cm^3^, respectively. The volume of the olfactory cilia that constitutes 10% of the olfactory epithelium is equal to (9.0 ± 0.5) × 10^−3^ cm^3^, and the volume of the respiratory cilia constituting 2% of the volume of respiratory epithelium is equal to (1.2 ± 0.08) × 10^−3^ cm^3^. The filtrate volume for all samples is 3.5 cm^3^. Knowing the filtrate concentrations and volume of the epithelia and cilia, the concentration of zinc nanoparticles in the experimental samples (C_s_) can be calculated as C_s_ = (V_f_/V_s_) × C_f_, where V_f_ is the filtrate volume, V_s._ is the sample volume, and C_f_ is the filtrate concentration. The concentrations of zinc nanoparticles in the olfactory and respiratory epithelia and cilia are shown in Table 3.

**Table 3:** Estimated concentrations of zinc nanoparticles in the epithelia and cilia.

Epithelium/cilia	Olfactory epithelium	Respiratory epithelium
^a^Epithelial filtrate, nM	0.27 ± 0.05	0.11 ± 0.05
Epithelium, nM	10.3 ± 2.5	7.9 ± 2.5
^b^Filtrate of cilia, nM	0.25 ± 0.05	0.36 ± 0.05
Cilia, µM	0.1 ± 0.025	1.0 ± 0.2

^a,b^Epithelial filtrates were diluted 50-fold in EOG experiments.

It should be noted that the concentration of zinc nanoparticles in the respiratory cilia is tenfold higher than that in the olfactory cilia.

### Endogenous zinc nanoparticles are elemental nonoxidized metals.

**Figure 2:**
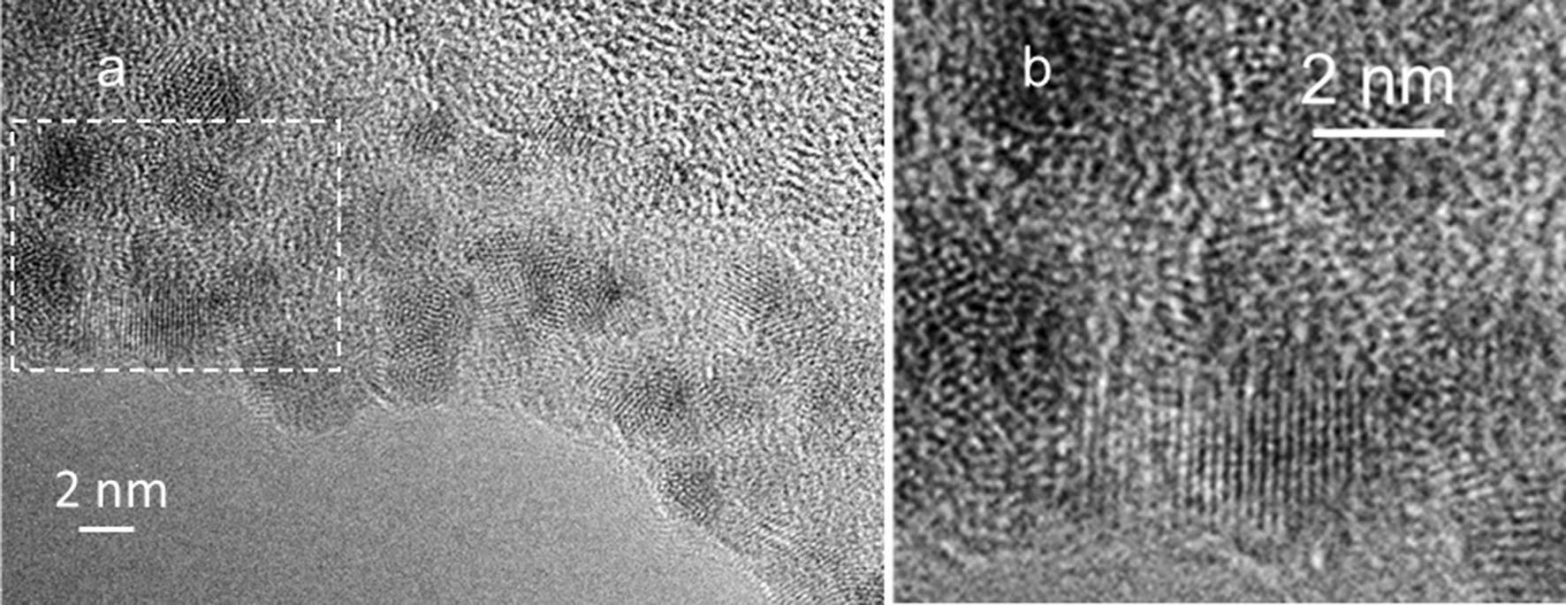
TEM micrograph of metal nanoparticles in a preparation of olfactory cilia. (**a**) Film was produced by drying a suspension of olfactory cilia containing metal nanoparticles. (**b**) Enlarged dotted area. The images represent 11 micrographs of nanoparticles in the samples of olfactory cilia.

The TEM micrograph of metal nanoparticles detected in a preparation of olfactory cilia is shown in Fig. 2.

**Figure 3:**
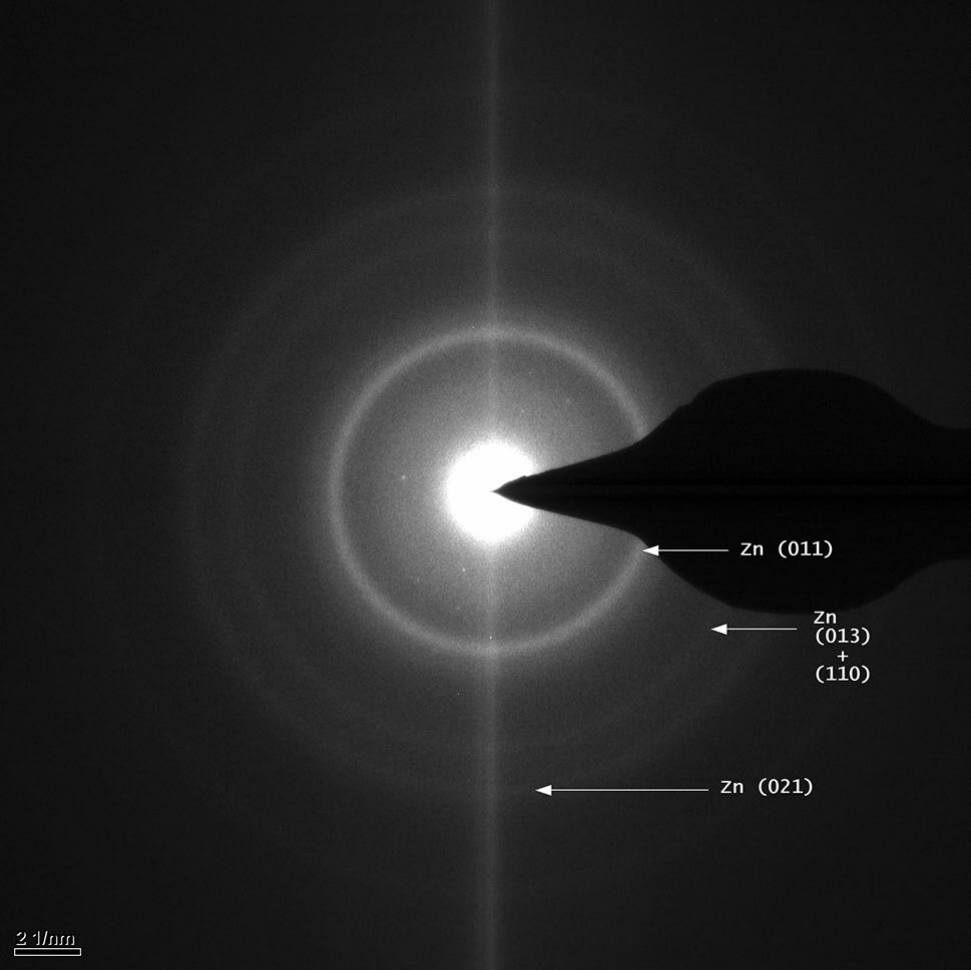
Selected area diffraction (SAD) pattern of zinc nanoparticles obtained from a preparation of olfactory cilia. The image is a representative of 16 SAD images of zinc nanoparticles in olfactory cilia.

The micrographs show nanoparticles 2–4 nm in diameter (Fig. 2a). The nanoparticles have crystal lattice fringes (Fig. 2b). The selected area diffraction (SAD) pattern of zinc nanoparticles obtained from a preparation of olfactory cilia is shown in Fig. 3.

The lines Zn (011), Zn (013) + (110), and Zn (021) are detected in the SAD patterns. The micrograph contains no oxidized zinc rings indicating the absence of oxidized zinc. Occasionally, some rings cannot be indexed (without identification of other constituents of the sample); however, there rings are rare. Complete absence of Zn (002) is detectable if present.

**Figure 4:**
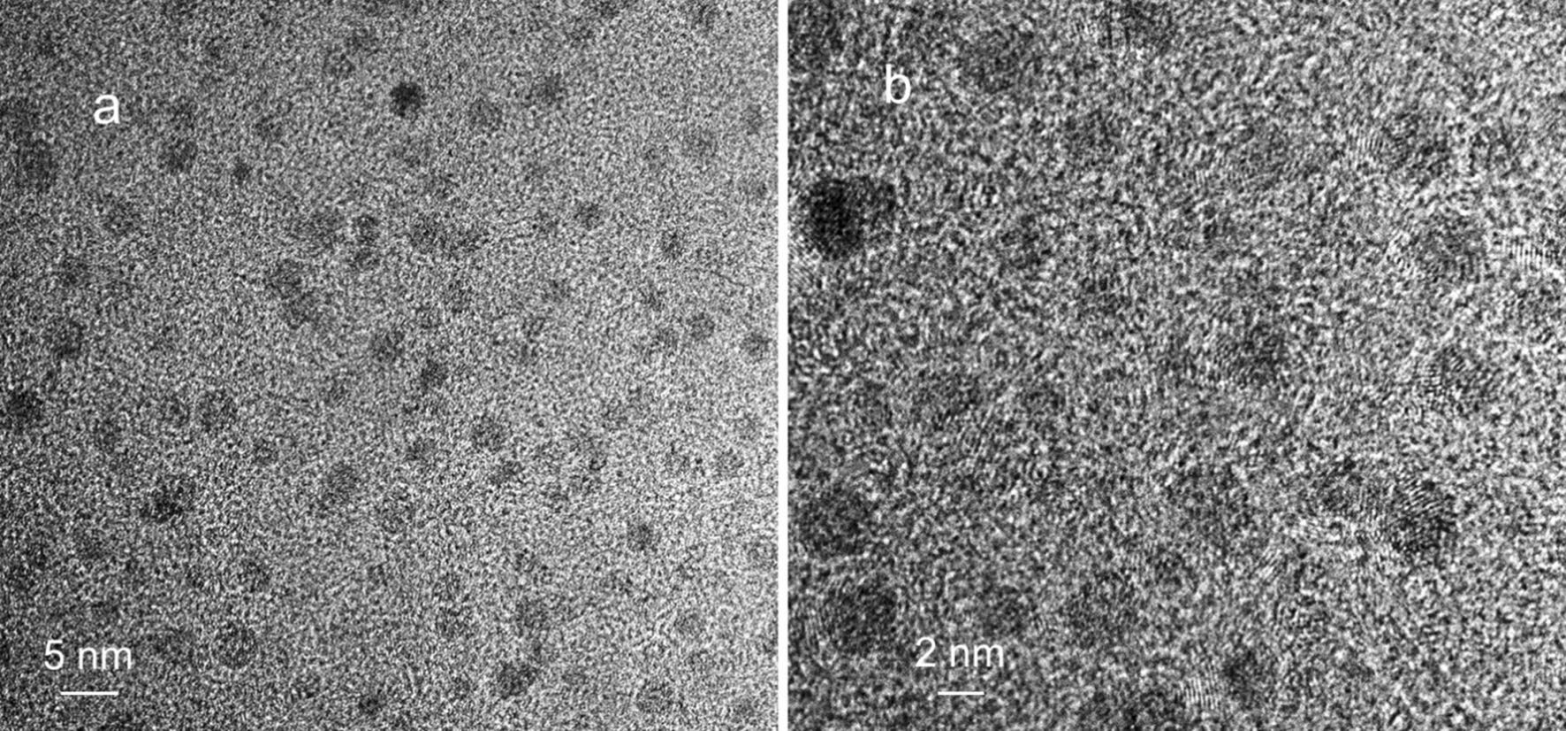
TEM micrograph of metal nanoparticles in a preparation of respiratory cilia. (**a**) Film was produced by drying respiratory suspension containing metal nanoparticles. (**b**) Enlarged area. The images are representative of 15 TEM micrographs of metal nanoparticles in the samples of respiratory cilia.

The TEM micrographs of metal nanoparticles in a preparation of respiratory cilia are shown in Fig. 4.

Metal nanoparticles 2–4 nm in size are shown randomly spread in the images. Figure 4b shows nanoparticles with visible crystal lattice fringes. (See also Supplementary Fig. S4).

**Figure 5:**
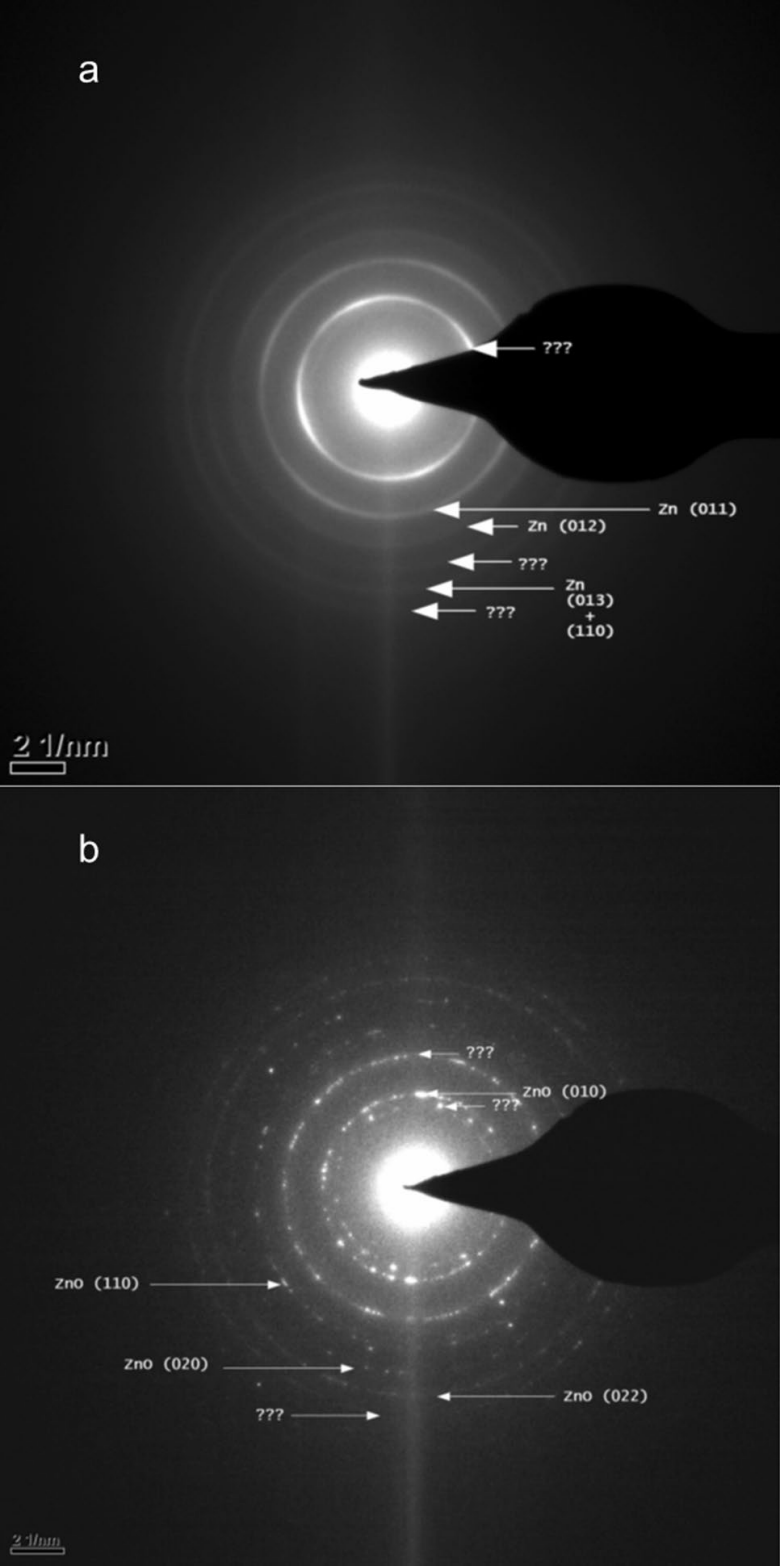
Selected area diffraction (SAD) pattern of zinc nanoparticles obtained from a respiratory preparation. (**a**) Freshly prepared sample. (**b**) The same sample after being exposed for 27 days to open air at room temperature. Each SAD pattern is a representative of 15 micrographs of metal nanoparticles in the samples of respiratory cilia.

The sample of respiratory cilia and olfactory cilia have no oxidized zinc (ZnO rings) (Fig. 5a).

The rings Zn (011), Zn (012), and Zn (013) + (110) can be detected in addition to the numerous rings that cannot be indexed suggesting that substantial amount of other constituents is present in addition to Zn. Complete absence of Zn (002) can be detected if present. An unidentified ring closest to the center is textured and has fourfold symmetry. When this sample was exposed to open air at room temperature for 27 days, a clear oxidized zinc pattern was detected including ZnO (010), ZnO (110), ZnO (020), and ZnO (022) lines (Fig. 5b).

## Discussion

We demonstrated that small elemental engineered zinc nanoparticles delivered in combination with an odorant considerably enhance the olfactory response to the odorant and do not influence the electrical responses of the nasal respiratory epithelium. The olfactory responses to the engineered zinc nanoparticles are dose-dependent and zinc-specific because other tested metal nanoparticles, including copper, silver, gold, and platinum, do not enhance the response. The enhancement of the odorant response by the engineered zinc nanoparticles was reproduced in Zhejiang University^16^. Rat olfactory epithelium was mounted on a microelectrode array and an electrical signal elicited by an odorant was substantially enhanced when the engineered zinc nanoparticles were added to an odorant.

The odorant response triggers the enzymatic cascade in the cilium (receptor, G-protein, adenylyl cyclase, ion channel, and phosphodiesterase). The G-protein is disengaged from the receptor to stimulate the generation of cyclic AMP by adenylyl cyclase; cAMP opens the ion channels to pass the ion current. cAMP is hydrolyzed by phosphodiesterase thus terminating the process (for review see^1^). High resolution fluorescence microscope images of the cilia detected vesicles, which presumably originate from the resealing of the cilia detached by sonication (Supplementary Fig. S3). The vesicle formation in the cilia preparations was confirmed by electron or light microscopy in various species^17−20^. Biochemical and electrophysiological studies indicate that the cilia preserve the active components of the enzymatic cascade^20−24^. The cilia preparation was shown to generate an ion current in response to odorant activation^18,25^. To preserve the functional enzymatic activity, the cilia preparation should have a closed compartment that includes the membrane-bound and cytoplasmic components of the cascade. The proteomic analysis of a cilia preparation demonstrated the presence of a fraction of soluble proteins originating from the cytoplasm^26^, including the cytoplasmic components of the enzymatic cascade^27^. Thus, the cilia preparation used in our study apparently preserves the cytoplasmic composition of the cilia, and zinc nanoparticles obtained from this preparation are a part of the intact cilia.

Zinc metal nanoparticles obtained from the olfactory and nasal respiratory cilia enhance the responses to an odorant similar to that generated by engineered zinc nanoparticles. Comparison of the peak electrical responses enhanced by the engineered zinc nanoparticles with those enhanced by the endogenous zinc nanoparticles enabled estimation of the levels of zinc nanoparticles in the olfactory and respiratory epithelia and cilia. The concentrations of zinc nanoparticles in the olfactory and respiratory cilia are 0.1 µM and 1.0 µM, respectively. Information on the distribution of zinc nanoparticles in animals is currently unavailable.

Zinc is an important element in neurobiology^9^. In the majority of the studies, the levels of zinc in the animal samples were analyzed by techniques that cannot differentiate between zinc ions and zinc nanoparticles^10^. Zinc is detected in a number of regions of the CNS; especially high levels of zinc are detected in the olfactory bulb (OB)^28^. The zinc concentration in the whole rat blood is ≈100 μM^29^, but Zn^2+^ ion content in the neuronal extracellular fluid is estimated to be 0.15 μM^30^. The intracellular free Zn^2+^ ion concentration in neurons is in the picomolar range and fluctuates within this range^31,32^. The physiological function of the endogenous Zn^2+^ ions in the olfactory sensory neurons is not described in the literature. However, the addition of zinc ions at 20 μM concentration inhibits the stimulatory GTP-binding protein of adenylyl cyclase^33^, an essential component of the initial events of olfactory signal transduction. The physiological function of endogenous Zn^2+^ ions has been extensively investigated in the neurons of the olfactory bulb. Zinc ions are localized in the synaptic terminals. Membrane depolarization releases zinc ions, and extracellular synaptic concentrations of zinc ions can be as high as 100 μM or even 300 μM. The released zinc modulates the excitability of the neurons^34^.

The endogenous zinc nanoparticles play a critical role in the electrogenesis of olfactory cilia. The molecules of an odorant interact with the olfactory receptors located in the ciliary membrane and initiate an electrical signal. Delivery of natural zinc nanoparticles to the receptors in combination with an odorant produces a substantial increase in the amplitude of the electrical signal^2^. The dose-dependency of zinc enhancement of the electrical signal suggests a certain stoichiometry of interaction of metal nanoparticles with the receptor. A single metal nanoparticle binds two receptor molecules to create a dimer^4^. A fraction of olfactory receptors of the cilia have been demonstrated to be in the dimeric state, while the rest of the receptors remain in the monomeric state^35^. Receptor dimers are critical because only dimeric receptors are functionally active and participate in signal transduction, and the monomeric receptors are passive^36^. The receptor monomers and dimers are in a dynamic equilibrium^37^. We hypothesize that endogenous zinc nanoparticles suspended in the cytoplasm of the cilia are in equilibrium with the nanoparticles that are bound to the receptor dimers in the ciliary membrane. An increase in the zinc level in the ciliary membrane or cytoplasm produces new dimers from free receptor monomers. The enhancement of the olfactory signal by zinc nanoparticles delivered in combination with an odorant has a simple explanation. The endogenous zinc nanoparticles generate a particular quantity of the operational receptor dimers that are activated by the odorant and participate in the initiation of the olfactory signal. The rest of the monomeric receptors remain passive and do not contribute to the odorant-evoked olfactory response. If the combination of natural or engineered zinc nanoparticles with the same odorant is delivered to the olfactory epithelium, new receptor dimers are formed from the monomeric receptors. The input of new receptor dimers will then increase the odorant-evoked olfactory response. An increase in zinc concentration enhances the conversion of the monomeric receptors into dimers until the monomers are depleted and the odorant-evoked olfactory response is saturated (as shown in Fig. 1c). Olfactory sensory neurons have a lifespan of approximately 60 days^38^ and are continuously replaced by younger neurons originating from the basal stem cells. Cilia of new olfactory neurons replenish zinc nanoparticle from the olfactory epithelium.

Additionally, zinc is a very important element in the respiratory epithelium. Zinc can be an antioxidant^39^, a membrane and cytoskeletal stabilizer^40^, an antiapoptotic agent (Zn protects the upper respiratory epithelial cells)^41^, an essential cofactor for DNA synthesis^42^, a component of wound healing^43^, and an anti-inflammatory agent^44^.

Zinc is involved in the essential properties of the respiratory cilia. Incubation of the mouse nasal septal epithelium in a zinc solution (50 µM ionic zinc) induced a fourfold increase in the ciliary beating frequency according to a high-speed digital video imaging^45^. In the rats, zinc was reported to influence the length of the bronchial cilia, the number of cilia per epithelial cell, and the number of intact epithelial cells^46^.

Current studied demonstrated high importance of zinc in olfactory and respiratory epithelia; however, the physical state of the metal has not been clear in all studies. Apparently, zinc exists in two main states in the mammalian body, the first of which is tightly bound to metalloproteins and zinc finger proteins and the second is a more labile ionic form that can be depleted by zinc deprivation^9^. In this study, the third state of zinc has been detected in the olfactory and respiratory cilia. The metal elemental crystalline zinc nanoparticles 2–4 nm in diameter were detected in the olfactory and respiratory cilia preparations. The specific diffraction pattern of nonoxidized zinc and the absence of oxidized zinc lines was observed in the samples of olfactory and respiratory cilia. The presence of zinc nanoparticles in the blood and olfactory and respiratory epithelia is significant and suggests a substantially broader presence of zinc nanoparticles in other cells and tissues.

Diffraction rings that are not zinc may belong to other unidentified elements that populate the diffraction pattern of the samples of the respiratory cilia. However, the presence of nonoxidized zinc nanoparticles in the respiratory cilia is strongly supported by TEM images that identified a zinc diffraction pattern. The oxidized zinc diffraction pattern was observed in the respiratory samples that were subjected to oxidation.

Complete absence of the zinc (002) diffraction line in the samples of olfactory and respiratory cilia may be important. The disappearance of allowed or appearance of forbidden diffraction lines were discovered by Renninger in 1937^47,48^. The phenomenon can be explained by multiple reflections. If a crystal is positioned in a way to produce a Bragg diffraction from a set of planes, another set of plains can be oriented to form the correct Bragg angle with the incident beam under certain circumstances. In this case, both diffracted beams exist in the crystal, and some interference of various diffraction channels may be detected. The intensity of a beam may disappear in the presence of another beam^47^.

Electron diffraction of nanocrystals is generally referred to as structural fingerprinting, which provides the standard method for identification of a crystal structure^49^. Therefore, the presence of the zinc diffraction pattern and absence of the oxidized zinc lines assure the elemental zinc state in the olfactory and respiratory samples. The TEM images and non-oxidized nature of the endogenous zinc nanoparticles compare well with those of the engineered Zn nanoparticles^5,6^. Our results are in agreement with the TEM and electron diffraction of the crystals of electrodeposited zinc^50^ and zinc nanowires^51^.

The presence of zinc nanoparticles in the olfactory and respiratory cilia is not unique. Using TEM and SAD, small zinc, copper, and iron crystalline nanoparticles were detected in the blood of humans, dogs, rabbits, and sharks^11^. The average concentrations of the metal nanoparticles in the blood of these animals were ~ 10 nM, which is consistent with the concentrations of the zinc nanoparticles in the olfactory and respiratory epithelia detected in the present study.

Relatively high concentrations of endogenous metal nanoparticles in the animal blood and tissues question the source of these particles. A number of plants, invertebrate animals, and microorganisms have the natural ability to reduce metal ions into the neutral atoms and assemble them into metal nanoparticles^51^. It is not known whether vertebrate animals have such ability. However, microorganisms of the gut microbiota can reduce metal ions into elemental metals. The reduction of zinc ions into metal zinc involves the reaction Zn^2+^ + 2e^-^ = Zn^0^, which is accomplished by electron transfer^4,51^.

Electron transfer occurs in mammalian gut, including human, mouse, rat, and guinea pig^52,53^. Therefore, the synthesis of zinc nanoparticles from zinc ions by gut microorganisms is a credible hypothesis. The validity of idea is supported by the fact that zinc uptake from the diet is controlled by the gut microorganisms^54^.

Dietary plants absorb zinc from the soil and are the critical source of zinc. Zinc is taken up by the roots from the soil primarily in the form of Zn^2+^ ions, which translocate through the xylem into the above-ground parts of a plant^55^. A fraction of the plant zinc ions is reduced into metal nanoparticles^56^. Thus, the plants serve as a source of zinc ions and zinc nanoparticles.

Our experimental results indicate that endogenous zinc nanoparticles are critical for the sensory functionality of the olfactory neurons. Certain criteria have to be met before the physiological significance of the endogenous zinc nanoparticles is accepted. These criteria are similar to those suggested for the validation that a given effector produces its action due to the activation of adenylyl cyclase^57^. The criteria are as follows: (i) the cilia of the olfactory sensory neurons contain zinc nanoparticles; (ii) the endogenous zinc nanoparticles modulate the function of the olfactory sensory neurons; and (iii) the modulation of the functions of the olfactory sensory neurons is not observed after the removal or replacement of zinc nanoparticles. Criterion (i) is supported by the present study; endogenous zinc nanoparticles were detected in the epithelium and cilia of the olfactory sensory neurons. Criterion (ii) is also supported by the experimental results of the present study that indicate a substantial modulation of the activity of the olfactory sensory neurons based on stimulation of an odorant response in the presence of endogenous zinc nanoparticles. Additionally, this criterion is validated by the fact that engineered zinc nanoparticles enhance odor-related brain activity in awake and anesthetized dogs^7^. Criterion (iii) is met since the enhancement of the odorant responses is not detected when engineered zinc nanoparticles are replaced by zinc ions^2^ and oxidized engineered zinc nanoparticles^5^ or removed by perfusion with ethylene glycol-bis(β-aminoethyl ether)-N,N,N′,N′-tetraacetic acid (EGTA)^58^. Thus, the physiological significance of endogenous zinc nanoparticles may be recognized considering these criteria.

The present study demonstrates the presence of zinc nanoparticles in the olfactory and respiratory cilia and shows their physiological significance. In the future experiments, we will profile other metal nanoparticles present in the cilia and examine their physiological functions. The mechanism of olfactory enhancement will be investigated.

Overall, our results suggest that metal nanoparticles coexist with metal ions and bound metals in mammals. However, new methods and approaches are required to test this conclusion. Metal nanoparticles are easy to harvest and study ex vivo under various conditions to evaluate transition to the human models and investigate the mechanisms of neuron enhancement and other processes of cell communication. If our conclusion is correct, the studies of metal nanoparticles in animals and humans may provide unexplored approaches to olfactory physiology and other areas of research and medicine where nanoparticles can be used for therapeutic purposes.

## Materials and Methods

All procedures were performed in accordance with relevant guidelines and regulations.

### Animals

The animal protocol was approved by the Auburn University Institutional Animal Care and Use Committee (AU IACUC). Adult male Sprague–Dawley rats (Envigo, Dublin, VA) weighing 200–220 g were used.

### Endogenous zinc nanoparticles derived from olfactory and respiratory cilia

The samples of olfactory cilia were prepared by a modification of the published procedures^21^. Rat olfactory epithelia from four adult males were rapidly dissected, pooled in 100 ml of ice-cold Hanks balanced salt solution (HBSS) without Ca^2+^and Mg^2+^ containing 2 mM EDTA, and sonicated by a Microson tip sonicator (Heat Systems, NY) at 10 W, 23 kHz, and 7 °C under nitrogen gas using 6 cycles of 10 s sonication and 10 s cool down. The deciliated epithelia were sedimented for 10 min on ice, and the supernatant containing the detached cilia was collected and centrifuged for 10 min at 9.0 × g at 4 °C. The pellet was resuspended and washed in 1 ml of PBS. After centrifugation for 7 min at 9.0 × g at 4 °C, the pellet was resuspended in 20 mL of PBS and gently vortexed for 1 min. Then, the suspension was consecutively filtered through 30 and 3 kDa cutoff membrane ultrafilters (Amicon; Millipore, Ireland) (Supplementary Fig. S6). The filtrate was centrifuged for 1 h at 87,000 × g at 8 °C (Supplementary Figs. S1–S3). The supernatant contained the suspension of endogenous zinc nanoparticles. This suspension was considered the cilia preparation and was used in EOG experiments for modulation of the odorant responses and in TEM experiments. The same procedures were used for preparation of the samples of the nasal respiratory epithelium.

### Endogenous zinc nanoparticles derived from olfactory and respiratory epithelia

Rat olfactory epithelia from four adult males were rapidly dissected and deciliated as described in the previous paragraph. The deciliated olfactory epithelia were pooled and incubated in 15 mL of ice-cold HBSS for 10 min. Then, the tissue samples were transferred in a glass-glass homogenizer; 2 mL of HBSS was added and the tissue was homogenized; the extract was consecutively filtered through 30 and 3 kDa cutoff membrane ultrafilters (Amicon) (Supplementary Fig. S6). After the filtration, the filtrate was centrifuged for 1 h at 87,000 × g at 8 °C. The supernatant containing suspension of endogenous zinc nanoparticles was used in EOG experiments. The same procedures were used for the samples of the nasal respiratory epithelium.

### Immunohistochemistry

#### Olfactory and respiratory epithelia

The samples of the olfactory and respiratory tissues were fixed in Bouin’s solution, placed in a cassette and paraffin infiltrated in a Tissue Tek VIP processor (Rankin Biomedical Corporation, MI, USA). The tissue was embedded in paraffin, and 6 µm sections were mounted atop glass slides. The sections were then deparaffinized in Hemo-De (Scientific Safety Solvents, TX USA). Subsequently, the sections were hydrated with graded ethyl alcohol series of 100, 95, 70, and 0% using distilled water. The sections were permeabilized in 0.1% Tween 20 (Sigma-Aldrich Japan Ltd) and humidified; the sections were blocked with 5% goat or donkey serum at room temperature for 1 h. Blocked sections were exposed to the following antibodies diluted in 5% goat or donkey serum in PBS: adenylate cyclase 3 (1:400, Invitrogen; PA5-35,382), anti-olfactory marker protein (1:50, Abcam; ab87338), and acetyl-alpha tubulin (1:50, ThermoFisher Scientific; 6-11B-1). The sections were thoroughly washed in a Coplin jar for 2 h and incubated in the dark with secondary antibodies (Alexa Fluor 488 or Alexa Fluor 555; 1:500; ThermoFisher Scientific) in the blocking buffer (5% goat serum) at room temperature for 1 h:. The slides were subsequently washed in a Coplin jar with PBS and 0.01% Tween-20, dehydrated, mounted with Eukitt mounting media (Sigma-Aldrich), and cover-slipped.

#### Cilia

Cilia were collected using the same procedure as described in section “Endogenous zinc nanoparticles from olfactory and respiratory cilia”; the samples were blocked with 5% goat or donkey serum at 4 °C for 1 h. The blocked cilia were centrifuged at 26,000 × g for 7 min at 4 °C. The resulting pellet containing the cilia was washed once in 500 µL of PBS. Then, the cilia were exposed to primary antibodies diluted in 5% goat or donkey serum in PBS (adenylate cyclase 3 (1:100, Invitrogen; PA5-35,382) and acetyl-alpha tubulin (1:50, ThermoFisher Scientific; 6-11B-1)) and incubated for 45 min at 20 °C. After the incubation, the cilia were centrifuged at 20,000 × g for 10 min at 4 °C. The pelleted cilia were washed twice with PBS and centrifuged again at 20,000 × g for 10 min at 4 °C. Then, the cilia were incubated in the dark with secondary antibodies (Alexa Fluor 488 or Alexa Fluor 555; 1:500; ThermoFisher Scientific) in the blocking buffer (5% goat serum) at room temperature for 40 min. The cilia were successively washed twice with PBS and 0.01% Tween-20 and centrifuged at 20,000 × g for 10 min at 4 °C. The resulting pellet of the cilia was resuspended in 100 µL of PBS and used for microscopy.

#### High-resolution fluorescence microscopy

The fluorescent optical system with a condenser^59^ was positioned in an Olympus BX51 microscope (Olympus America Inc., PA, USA). A light source (EXFO120, Photonic Solution, Inc., Canada) was linked to the condenser using a liquid light guide through a dual mode fluorescent filter [87]. The samples were observed by using an Olympus 100X UPlanApo oil iris objective and fluorescence quad filters, Chroma 89,101 × and 89101 m (Chroma Technology Corp., VT, USA); the images were recorded with a Zeiss AxioCam camera (Carl Zeiss Inc., NY USA). Resolution of the images was 90 nm achieved using an optical illumination system with a high-aperture cardioid annular condenser^59^. The dual mode optical system allows simultaneous recording of the fluorescent and high-resolution darkfield images^60^.

#### Concentrations in the filtrate of the cilia and epithelia

To estimate the concentrations of the endogenous nanoparticles in the filtrates from the olfactory and respiratory epithelia and cilia, a calibration curve was obtained by recording the responses to an odorant at the known concentrations of engineered zinc nanoparticles (Fig. 1f).

#### Odorants

Odorants were obtained from Sigma-Aldrich. An odorant mixture of ethyl butyrate, eugenol, and ( +) and (–) carvone in water was prepared with a vortex mixer and stored in a dark glass container at 283 K (5 °C). This odorant mixture was identical to that used in our previous publications^2,4−8^. The spatial clustering of the principal responses to the individual odorants of this mixture was shown to be statistically distinct and characterized by variable glomerular patterns^61^; for simplicity, the term “odorant” is used to described the mixture of odorants in the text.

#### Electrophysiological recording

Electrophysiological recording was described in our previous publications (for example, in^2^). In brief, rat olfactory epithelium (or nasal respiratory epithelium) was dissected and positioned in a perfusion chamber to immerse the basal parts in the buffer solution (137 mM NaCl, 5.3 mM KCl, 4.2 mM NaHCO_3_, 0.4 mM KH_2_PO_4_, 3.4 mM Na _2_HPO_4_, 5.6 mM D-glucose, 0.8 mM MgSO_4_, and 1.2 mM CaCl_2_, pH 7.4), whereas the olfactory (or respiratory) cilia were positioned in the water/air interface. The EOG recording glass electrodes were linked through an Ag/AgCl wire to an amplifier to record the signals from OE. After the connection between the electrode and OE was established, an air pulse of the odorant mixture was applied, and a continuous electrical signal was recorded as a function of time. Odor-activated current-clamped voltage responses were recorded as EOG with an integrating patch-clamp amplifier (MultiClamp 700A Amplifier, Molecular Devices). The analog signal was filtered at 0–5 kHz and digitized using a digital converter (DigiData 1322 A). Data acquisition, storage, and subsequent analysis were carried out using the pCLAMP software (Axon Instruments) and exported in ASCII format for additional analysis. The recording chamber was enclosed in a grounded Faraday box on a vibration isolation table (GS-34 Newport, Newport Corporation, Franklin, MA, USA). The odorant vapor was produced by a homemade olfactometer^62^ that was used for precise computer-controlled delivery of predetermined quantities of the odorants over a programmed time interval. For stimulation purposes, a 0.25 s pulse of the odorant mixture was formed by a computer-controlled pneumatic PicoPump PV800 (World Precision Instruments, Sarasota, FL, USA). A pulse of positive pressure drove the odorant into a glass nozzle directed at the olfactory epithelium. There is a variability in the EOG amplitudes measured at various contact points of olfactory epithelium from various animals. However, the correctly normalized relative EOG values are conserved between a single animal and various animals of the same age and breed^6^.

#### Engineered zinc nanoparticles

Preparation and characterization of engineered zinc nanoparticles were reported previously^5,6^. In brief, engineered zinc nanoparticles were prepared by a high-voltage electrical discharge method^63^. The system consisted of a water container with a high voltage generator connected to two metal electrodes submerged in water. Controlling the voltage and distance between the electrodes produces an underwater plasma that creates a very fine dispersion of a metal into nanoparticles. Suspended particles are separated from the sediment and subjected to centrifugation at 15,000 × g for 2 h at 25 °C. After centrifugation, the pellet was discarded, and the supernatant was subjected to additional centrifugations to produce fractions of the nanoparticles enriched in particles of particular sizes. The physical properties of the particles were analyzed by electron microscopy, atomic force microscopy, X-ray photoelectron spectroscopy, and laser Doppler velocimetry. The total concentration of the metal in the suspension was measured by atomic absorption spectra (GTW Analytical Services, Memphis, TN, USA). The typical concentration of zinc in stock suspensions was ~ 4 µg/L. Zinc nanoparticles were distributed from 1 to 4 nm in diameter with a median of 1.2 nm^5^. The particles were round and crystalline. The crystal structure of metallic zinc has a hexagonal close-packed lattice with a constant a = b = 0.266 and c = 0.495 nm. The unit cell contains 6 atoms and has a volume of 0.0912 nm^3^. The total number of atoms in a 1.2 nm zinc nanoparticle was estimated to be 59. The number of atoms in the core and shell were 12 and 47 atoms, respectively^5^. These numbers of atoms are in close agreement with the “magic number” full-shell nanoparticles with 13 and 55 atoms, when each metal atom has the maximum number of the nearest neighbors, which imparts a degree of extra stability to the full-shell nanoparticles^64^. We found that freshly prepared engendered zinc nanoparticles at the age of 1 day stored at 5 °C in water containing 97% of metal zinc atoms and 3% of ZnO^6^. After 317 days at this temperature, the nanoparticles oxidized only by 1% and had 96% of metallic zinc. However, freshly prepared zinc nanoparticles were able to enhance olfactory response to an odorant for 60 days. Engineered zinc nanoparticles covered by a thin layer of polyethylene glycol maintained olfactory enhancement for over 300 days^6^. Stable suspensions of small nanoparticles of Zn, Cu, Ag, Au, and Pt were the only suspensions we were able to produce in our laboratory.

#### TEM and electron diffraction

TEM was carried out using an FEI Titan (NanoFab Microscopy. Gaithersburg, MD, USA) operated at 80 kV and 300 kV. Small drops of the cilia preparation were deposited onto a QUANTIFOIL1Holey carbon film on copper TEM grids. All images were acquired in the TEM mode at 300 keV. All electron diffraction results were acquired in the selected area diffraction (SAD) mode at 300 keV with a 10 µm SAD aperture. Diffraction assessment was based on Zn in the hexagonal close-packed (hcp) phase (space group P 63/m c, ICSD #64,990) and ZnO in the hcp phase (space group P 63 m c, ICSD #26,170). Samples were prepared by pipetting the drops of the cilia preparation onto a lacey-carbon TEM grid at room temperature and were air dried one day before the analysis.

### Statistical analysis

Data averaging, curve fitting, and graph plotting were performed using Origin 2019 (Northampton, MA, USA) and 2010 Microsoft Excel.

### Disclaimer

Certain commercial equipment, instruments, or materials are identified in this paper to specify the experimental procedure adequately. Such identification is not intended to imply recommendation or endorsement by the National Institute of Standards and Technology, nor is it intended to imply that the materials or equipment identified are necessarily the best available for the purpose.

## Supplementary information

Supplementary Information.
