# Supplementary material for: Endogenous zinc nanoparticles in the rat olfactory epithelium are functionally significant
Source: Sci Rep. 2020 Oct 28;10:18435. doi: 10.1038/s41598-020-75430-w (PMC7595131; doi:10.1038/s41598-020-75430-w)
Supplement: Supplementary file 1 — Supplementary Information. [file 41598_2020_75430_MOESM1_ESM.pdf]

Supplementary information

Endogenous zinc nanoparticles in the rat olfactory epithelium are functionally significant

Melissa Singletary, June W. Lau, Samantha Hagerty, Oleg Pustovyy, Ludmila Globa, and Vitaly Vodyanoy*

Membrane preparation from rat olfactory and respiratory epithelia and cilia

In the first step, we carefully dissected epithelia from a rat nasal cavity. To inspect the quality of the tissue and the presence of cilia we examine localization of the olfactory marker protein (specific marker of the olfactory sensory neurons. Fig. . S1 shows intact olfactory neurons (Fig. S1a, 1), knob of olfactory neuron and cilia (Fig. . S1a, 2), and basal cells (Fig. . S1a, 3). The respiratory epithelium features the characteristic cilia (Fig. S1b, 1) and basal bodies (Fig. S1a, 2). Both, olfactory and respiratory cilia contain tubulin. Therefore, tubulin antibodies are the best to visualize the olfactory and respiratory cilia

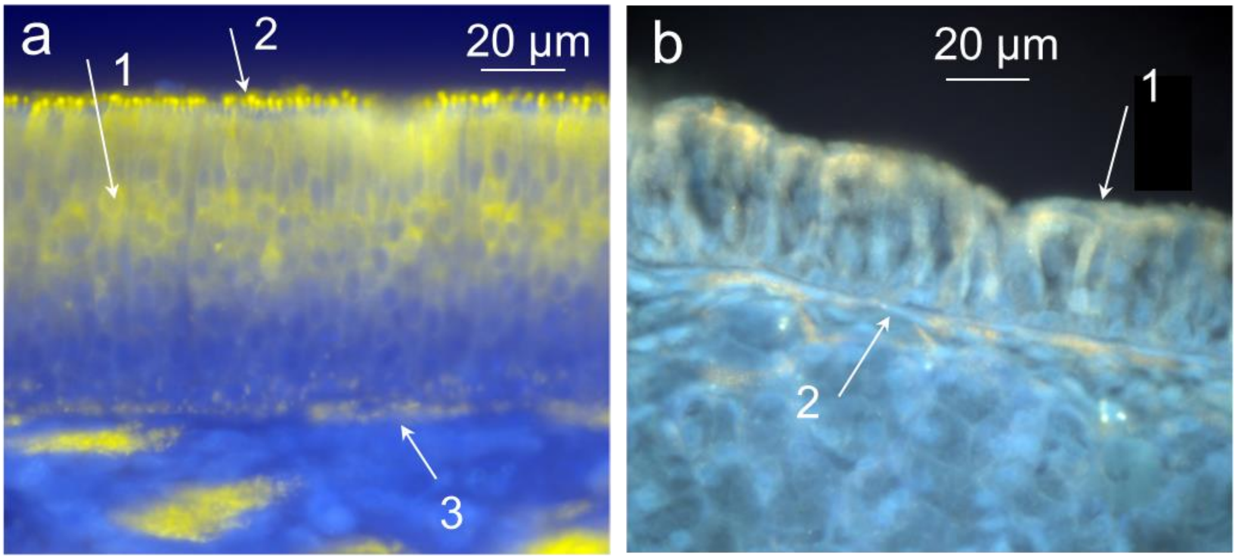

Fig. S1. Immunohistochemical localization of OMP in the olfactory and respiratory epithelia of rat. (a). Olfactory epithelium. 1. Olfactory sensory neuron. 2. Knob of olfactory neuron and cilia. 3. Basal cells. (b). Respiratory epithelium. 1. Cilia, 2. Basal bodies.

Fig. S2 demonstrates presence of cilia in both olfactory and respiratory epithelia.

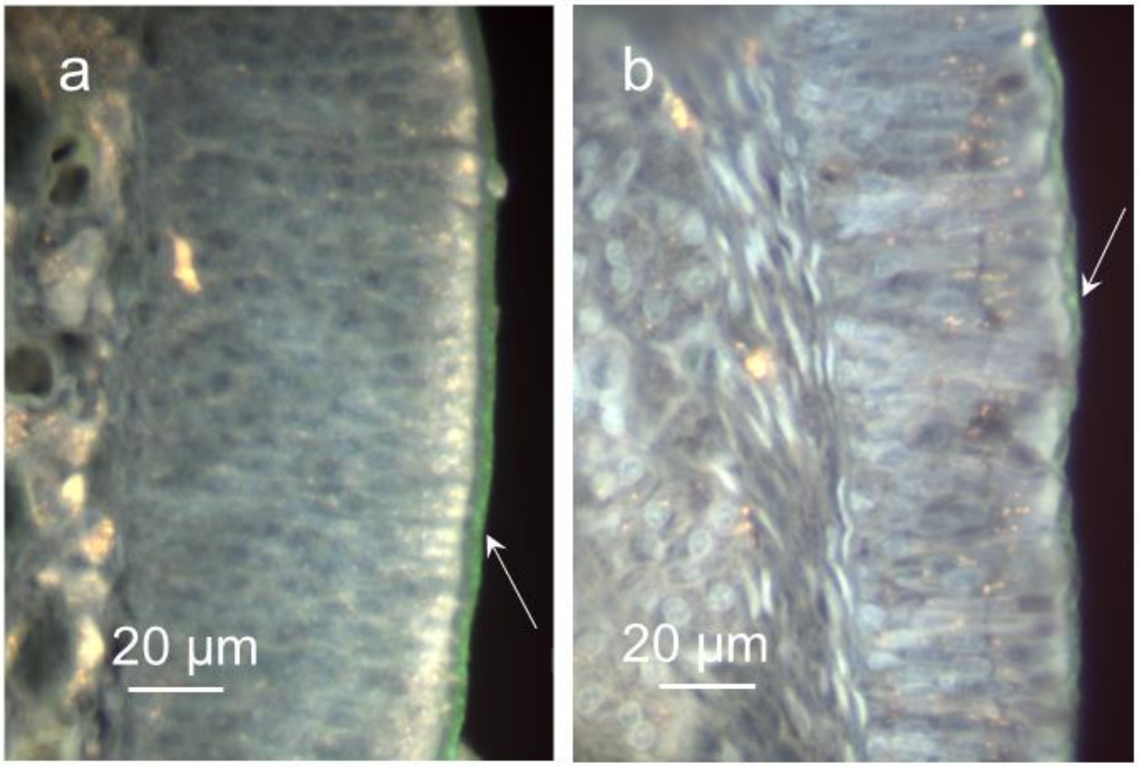

Fig. S2. Immunohistochemical localization of tubulin in cilia of the rat olfactory and respiratory epithelia. (a). Olfactory epithelium. 1. Olfactory cilia (arrow). (b). Respiratory cilia (arrow).

The next few steps includes further purification by sedimentation and series of centrifugation and washing of the cilia fraction that results in the cilia preparation shown in the Fig. S3. Fig. S3a illustrate single wall vesicles that are resulted in resealing and reshaping of detached cilia. We speculate that a single vesicle represents a single cilium, because a volume the vesicle is approximately equal to the volume of the olfactory cilium. The ultra-filtering the cilia preparation through 30- and 3 kDa filters and the consecutive high-speed centrifugation produce a suspension zinc nanoparticles originated from the cilia preparation.

The respiratory epithelium cilia are four times shorter than olfactory cilia, therefore respiratory cilia produce the much smaller and less optically resolved vesicles (Fig. S3b). Some respiratory cilia do not assume a vesicle shape and remain to be of a linear structure.

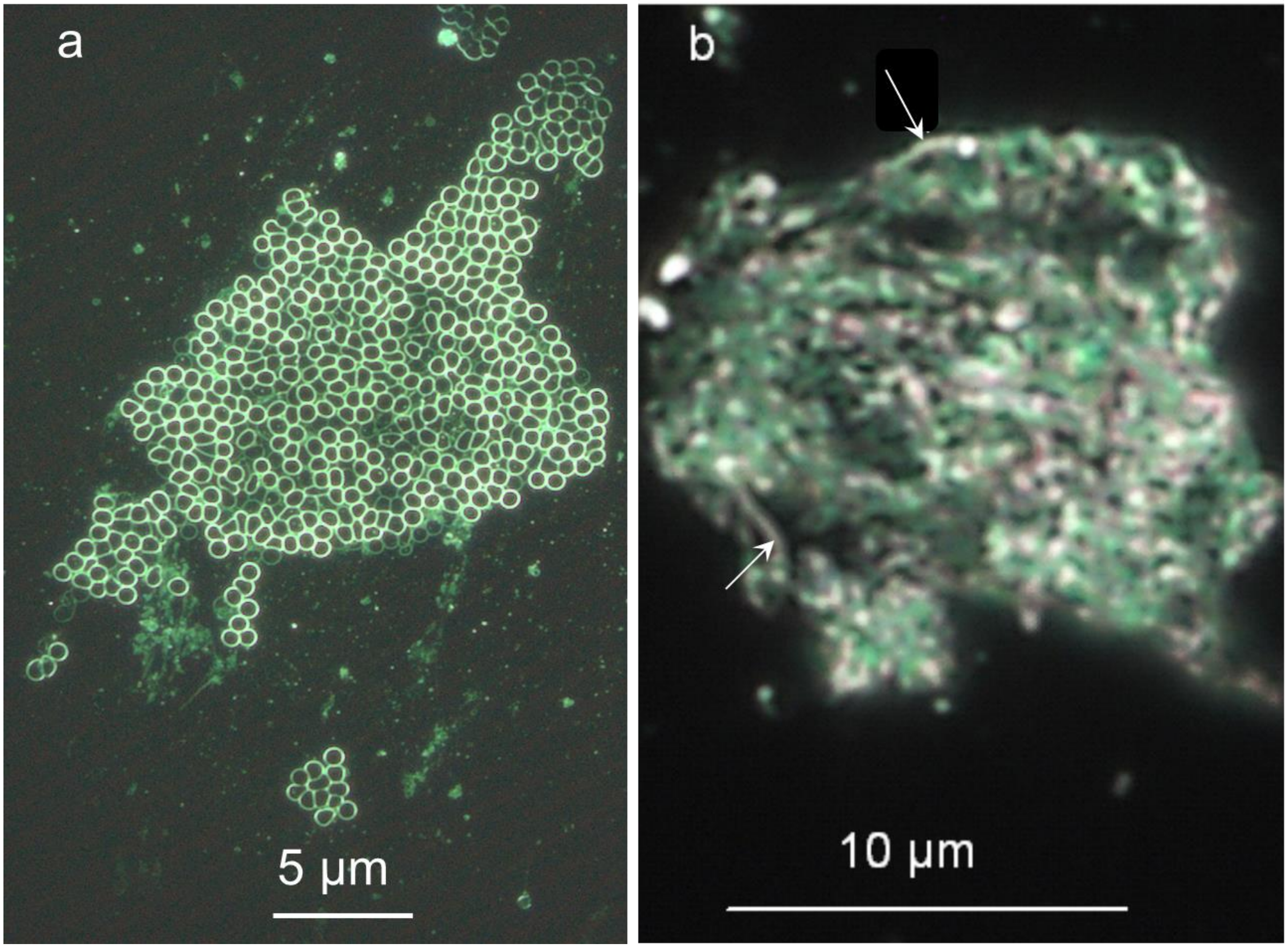

Fig. S3. Immunofluorescence of olfactory (a) and respiratory (b) cilia preparations stained with anti-tubulin antibodies. Arrows show pieces of respiratory cilia.

Electron microscopy of metal nanoparticles in olfactory cilia preparation

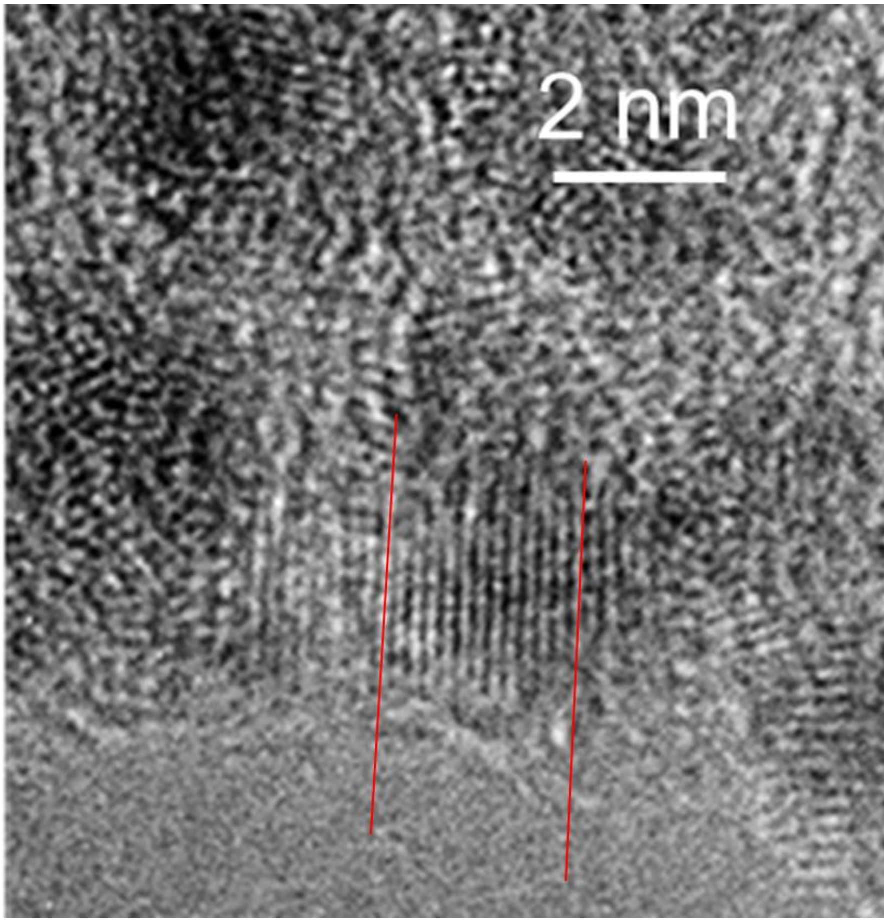

Fig. S4. TEM micrograph of metal nanoparticles in olfactory cilia preparation, Enlarged fragment of Fig. ure 2. The area between red lines shows distinct crystal fringes of 0.17 nm.

One can argue that the fringe pattern may result from crystallization of the salts presented in the buffer solution in the course of drying. It is difficult of ruling this out. A different interpretation is that 3-dimensional particles overlap in a 2D image, and some particles stick out in the stack, causing it to look like there is materials in the spaces in between. Furthermore, the fringes of 0.17 nm corresponds to the [0112] direction for the hexagonal close-packed crystal lattice of metal zinc[1].

Geometry of ciliated epithelium

The epithelium is approximated as a rectangular parallelepiped. Volume of Rectangular Parallelepiped = Surface Area × Height. Thus the volume of the olfactory and respiratory epithelia is calculated by the equation, $V = A \times T$, where A and T are the epithelial surface area and thickness, respectively. The cilia can be presented as a cylinder. Volume of a cylinder = Base Surface × Length. Thus the volume of a single cilium $(v) = (\pi d^2/4) \times l$, where d is a cilia diameter,

l is a cilia length. The volume of the olfactory and respiratory cilia, Vc=v×D×n, where A – an epithelium area, D – a density of ciliated cells, and n is the number of cilia per cell.

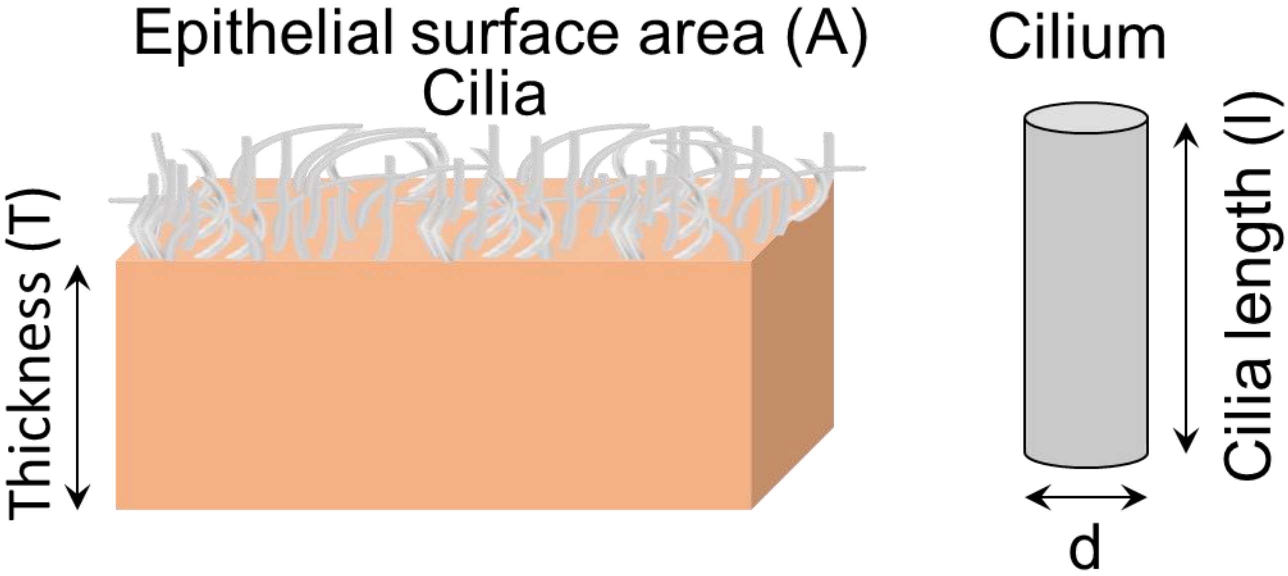

Fig. S5. Geometry of ciliated epithelium. A simplified structural diagram of ciliated epithelia shown in Fig. S1.

Ultrafiltration

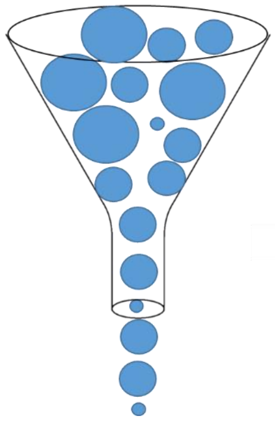

Molecular weight cut-off (MWCO) is a method that is defined as the lowest molecular weight (in Daltons) at which greater than 90% of a solute with a known molecular weight is retained by the membrane[2]. We use the consecutive filtration by 30 kDa and 3 kDa as a part of purification of cilia and epithelia preparations to remove 90% of proteins larger than 3 kDa and allow nanoparticles of 1-5 nm to pass to filtrate.

Fig. S6. A schematic diagram of ultrafiltration with 30- and 3-kDa Amicon Ultra filters.

References

1 Hagerty, S. *et al.* After oxidation, zinc nanoparticles lose their ability to enhance responses to odorants. *BioMetals* **29**, 1005-1018, doi:10.1007/s10534-016-9972-y (2016).

2 Erickson, H. P. Size and shape of protein molecules at the nanometer level determined by sedimentation, gel filtration, and electron microscopy. *Biol. Proced. Online* **11**, 32-51, doi:10.1007/s12575-009-9008-x (2009).
